# Microneedling Therapy in Atrophic Facial Scars: An Objective Assessment

**DOI:** 10.4103/0974-2077.53096

**Published:** 2009

**Authors:** Imran Majid

**Affiliations:** *Cutis Skin and Laser Clinic, Govt Medical College, Srinagar, India*

**Keywords:** Atrophic scars, microneedling, therapeutic results

## Abstract

**Background:**

Atrophic facial scars are always a challenge to treat, especially the ones that are deep-seated and/or involve much of the face. Microneedling or dermaroller therapy is a new addition to the treatment armamentarium for such scars that offers a simple and reportedly effective management of these scars.

**Aims:**

The aim of the present study was to perform an objective evaluation of the efficacy of dermaroller treatment in atrophic facial scars of varying etiology.

**Materials and Methods:**

Thirty-seven patients of atrophic facial scarring were offered multiple sittings of microneedling (dermaroller) treatment and their scars were evaluated and graded clinically and by serial photography at the start as well as at two months after the conclusion of the treatment protocol. Any change in the grading of scars after the end of treatment and follow-up period was noted down. The patients were also asked to evaluate the effectiveness of the treatment received on a 1-10 point scale. The efficacy of dermaroller treatment was thus assessed both subjectively by the patients as well as objectively by a single observer.

**Results:**

Overall 36 out of the total of 37 patients completed the treatment schedule and were evaluated for its efficacy. Out of these 36 patients, 34 achieved a reduction in the severity of their scarring by one or two grades. More than 80% of patients assessed their treatment as ‘excellent’ on a 10-point scale. No significant adverse effects were noted in any patient.

**Conclusions:**

Microneedling therapy seems to be a simple and effective treatment option for the management of atrophic facial scars.

## INTRODUCTION

Scarring is a particularly distressing phenomenon and is most unwelcome when it occurs on the face. Scars can arise on the face due to a number of causes, the commonest of which is acne vulgaris. Post-acne facial scarring is a psychologically devastating condition and the affected patient invariably suffers from low self-esteem and many other psychological ill-effects because of this condition.[1] Facial scarring has always been a challenge to treat and there are different treatment options for the management of these scars. However, the majority of these treatment options suffer from the limitation of either being marginally effective or else having considerable morbidity. Treatment options like laser resurfacing or dermabrasion that offer significant improvement in facial scars are invariably associated with considerable morbidity and downtime interference with the daily activities of the patient in the post-treatment period.[2,3] On the other hand, treatments like microdermabrasion and non-ablative resurfacing with lasers that are associated with a minimal or no downtime, do not show the same level of efficacy as the traditional, ablative resurfacing techniques.[4,5] New treatments and techniques are being added over the last few years to overcome these limitations.

One such device is dermaroller. Treatment with these hand-held devices is known by many names like microneedling therapy, collagen induction therapy or dermaroller therapy. There are some pathological as well as clinical studies now available in the world literature that have documented a favorable clinical and histopathological response in the skin after dermaroller treatment.[6,7] However, there is a definite paucity of objective clinical trials on the efficacy of dermaroller treatment in facial and other types of scars.

Post-acne facial scars have been classified into many morphological types and the ideal treatment option depends upon the type of scarring. Recently, a clinical grading system has been devised to grade the severity of post-acne facial scars [Table 1]. This grading system, proposed by Goodman and Baron, encompasses all the morphological types of post-acne scars and uses a simple clinical examination as the tool to grade the scars on objective lines.[8] This grading system can also be used to assess the severity of other etiological types of facial scars. The present study is aimed at ascertaining the efficacy of dermaroller treatment objectively in the management of atrophic facial scars.

**Table 1:** Grading of atrophic scars

Grade of atrophic scars	Clinical picture
**Grade 1**	Macular erythematous, hypo or hyperpigmented scars
**Grade 2**	Mild atrophy not obvious at social distances of >50 cm or easily covered by facial makeup or beard hair
**Grade 3**	Moderate atrophy obvious at social distances of >50 cm; not easily covered by makeup or beard hair, but able to be flattened by manual stretching
**Grade 4**	Severe atrophy not flattened by manual stretching of skin

## MATERIALS AND METHODS

The present study was performed on 37 patients suffering from localized or generalized atrophic facial scarring of variable etiology. The patients were photographed and assessed clinically at the time of enrolment to grade the severity of scarring, by a single trained dermatologist as per the grading system proposed by Goodman and Baron. Only patients with Grade 2 to Grade 4 atrophic scarring were enrolled for the study. A history of application of topical retinoids, use of systemic retinoids or any other scar treatment procedure in the previous three months were used as exclusion criteria. Presence of any active infection anywhere, active acne on the face or a keloidal tendency in the patient also served as exclusion criteria. Informed written consent was obtained from all the patients who were enrolled for the study. Microneedling or dermaroller treatment was performed at monthly intervals till a satisfactory outcome was achieved or a maximum of four sittings whichever was earlier. A minimum of three treatment sessions was considered essential for inclusion for assessment. Area of interest was anesthetized using a thick application of topical anesthetic cream (eutectic mixture of prilocaine and lignocaine), about 30-45 min before the procedure. Dermarollers with 1.5mm long needles were used and the endpoint for any treatment session was the presence of uniform bleeding points over the scarred area. In patients with deep-seated scarring, the skin was stretched in a perpendicular direction to the dermaroller movement so that the base of the scars could also be reached. After the end of the treatment regimen, the scars were again assessed and graded by the same trained dermatologist, and the patients were followed up monthly for the next two months. The final assessment and grading of scars was done at the end of two months of follow-up and repeat photographs were then taken. The appearance and grading of scars was then compared with that in the pre-treatment period and any change in the grading of scars was noted. In addition to this objective assessment, the change in the severity or grading of scars was assessed photographically also. On objective lines, an improvement of scarring by two grades or more was labeled as ‘excellent’ response while a ‘good’ response meant an improvement by a single grade only. In those patients where the scar grading remained the same after the completion of treatment irrespective of any visible change in the facial scarring the response was labeled as ‘poor’.

The patients were also given a preformed questionnaire at the end of the follow-up period wherein they were asked to rate the improvement in their scars on a 10-point scale. Rating above 6 was graded as ‘excellent response’, rating between 4 and 6 served as ‘good response’ and rating below 4 meant a ‘poor response’. A subjective assessment of the treatment protocol on the part of the patients was also thus obtained.

Adverse effects that arose as a result of the treatment were also noted down including any adverse effects in the immediate post-treatment period. The patients were specifically asked about the immediate post-treatment sequelae and whether there was any interference with their daily activities in the post-treatment period. Post-procedure, patients were advised topical antibiotic application for two to three days and sun avoidance for at least a week after each dermaroller treatment.

## RESULTS

**Figure 1:**
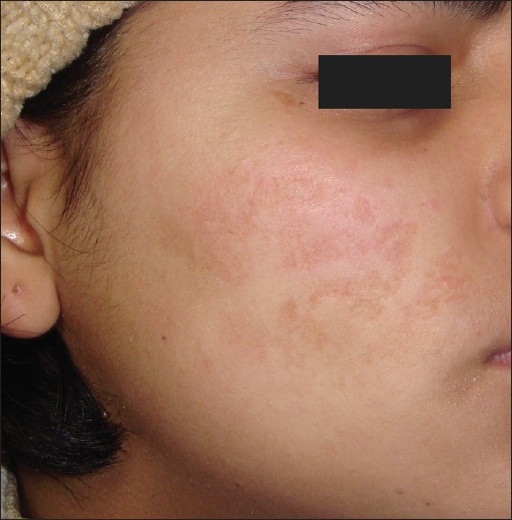
Post-herpetic scarring before treatment

**Figure 2:**
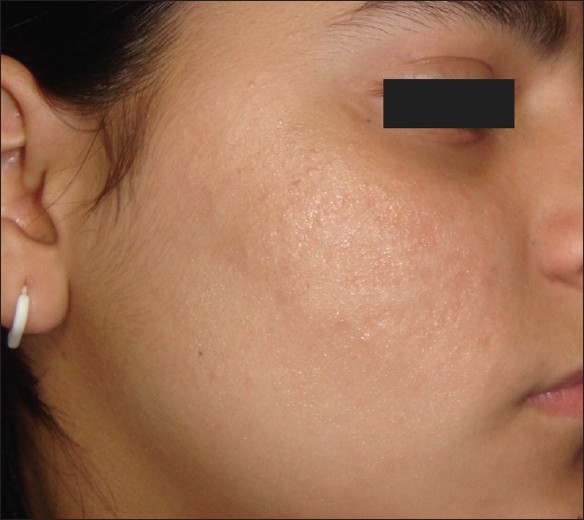
Post-herpetic scarring post-treatment

Of the 37 patients, the majority (32) were suffering from post-acne atrophic facial scarring while two patients each had post-traumatic and post-varicella scarring and one patient had post-herpetic scarring. Females (23) predominated over males (14) in the study group. The age of the patients ranged from 13 to 34 years, with the mean age of 22.4 years. The youngest patient was a female aged 13 years who was suffering from severe post-herpetic scarring [Figures 1 and 2] and the oldest patient was a male aged 34 years with severe post-acne scarring. The majority of the patients were however in the third decade of their life.

All patients tolerated the procedure well and except for a temporary erythema and post-inflammatory hyperpigmentation in one patient, no adverse effects were noted in any patient. No patient reported any interference in his/her daily activities in the immediate post-treatment period and the only change noted in the immediate post-treatment period was a mild crusting that persisted for one or two days. The patients were able to attend their daily duties on the same day or the next day after the dermaroller sitting.

Only one patient dropped out of the study as she could not complete the minimum three sittings required for final assessment The rest of the 36 patients were thus available for evaluation of results at the end of the study period.

Of the 36 patients who filled the questionnaire at the end of the study period, 29 patients reported the response as ‘excellent’ (7-10 on the 10-point scale), four patients reported the response as ‘good’ (score of 4-6) and only three patients reported the response as ‘poor’ (score of <4).

Objective assessment of the patients' scarring at the start of the study revealed Grade 4 scarring in seven patients, Grade 3 scarring in 21 patients and Grade 2 scarring in nine patients. The patient who dropped out of the study was suffering from Grade 4 scarring and therefore we had only six patients from the original Grade 4 scarring group for evaluation at the end of the study period. Of these six patients, only one patient achieved an ‘excellent’ response on objective assessment. In this patient the grading of scars at the end of treatment could be reduced to Grade 2. In three others, the scar grading could be reduced to third grade and thus the response in these patients was labeled as ‘good’. In two patients, no significant change could be observed in the severity of scarring and the response was thus labeled as ‘poor’.

In 21 patients with Grade 3 scarring, an excellent response was similarly noted in 16 patients (reduction to Grade 1 or less), three patients achieved a good response while two patients had a poor response to treatment.

In patients with Grade 2 scars all nine showed an excellent response to treatment.

Thus, overall, 26 out of the total of 36 patients (72.2%) showed an excellent response to dermaroller treatment while six others achieved a good response (16.7%). Only four patients (11.1%) out of the total of 36 failed to show a significant response to treatment [Table 2].

**Table 2:** Response to dermaroller treatment

Grade of scars	Number of patients	Excellent response	Good response	Poor response
**Grade 4**	6	1 (16.7)	3 (50)	2 (33.3)
**Grade 3**	21	16 (76.2)	3 (14.3)	2 (9.5)
**Grade 2**	9	9 (100)	0	0
**Total**	36	26 (72.2)	6 (16.7)	4 (11.1)

Figures in parenthesis are in percentage

Correlating the response rate with the grade of scarring present, an excellent response rate was achieved in the majority of patients with Grade 2 and 3 scarring. However, in patients with Grade 4 scarring, only one out of the six patients had excellent response, while in three others (50%), the response was good. However, because of only a few patients with Grade 4 scars in the present study, the comparative results could not be tested for their statistical significance.

**Figure 3:**
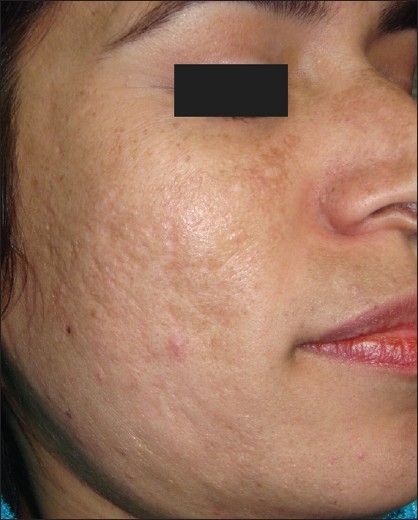
Post-acne scarring before treatment

**Figure 4:**
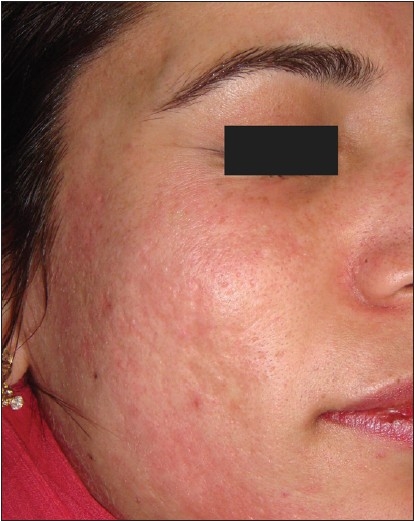
Post-acne scars after dermaroller treatment

Correlating the response with the morphological type of scarring present, we found a good to excellent response in rolling and boxcar scars while pitted scars showed only moderate improvement. However, contrary to expectations, we could observe a definite improvement in pitted scars and deep boxcar scars as well [Figures 3 and 4]. Deep tunnels and other complicated scars showed a poor response to treatment.

Of the five patients who had scarring from causes other than acne, we observed an excellent response in the single patient of post-herpetic scarring. This patient who had a Grade 3 scarring at the start of treatment [Figure 1] and we were able to reach a stage of Grade 1 scars at the end of treatment [Figure 2]. All the four patients of post-varicella and post-traumatic scarring (two each) showed a ‘good’ response to treatment on objective analysis.

## DISCUSSION

Microneedling therapy, also known as collagen induction therapy, is a recent addition to the treatment armamentarium for managing post-acne scars. The treatment is performed as an office procedure after application of a local anesthetic cream, by means of an instrument known as a dermaroller. A dermaroller is a simple, hand-held instrument consisting of a handle with a cylinder studded all around with fine, stainless steel needles 0.5 to 2 mm in length. This needle-studded cylinder is rolled on the skin in multiple directions to achieve a therapeutic benefit and hence the name ‘dermaroller’. These needles cause small pinpoint injuries on the treated skin, which apparently heal within two to three days with no post-treatment sequelae. Treatment with dermaroller is performed at four to eight week intervals and multiple sittings are needed to achieve the desired effect on the skin. Microneedling or dermaroller treatment is becoming popular all over the world, not only in the management of post-acne scars but also as an anti-aging therapy. There are certain advantages with dermaroller or microneedling therapy over laser resurfacing; former does not lead to any epidermal injury as is seen with lasers, there is minimal downtime associated with the procedure unlike ablative laser resurfacing and the treatment is far cheaper as compared to lasers. The treatment can be performed in an office setting and does not need any extensive special training or expensive instruments.

Choice of treatment of post-acne scars depends both on the morphological type as well as the severity of each scar present on the face.[9,^10^] Post-acne scars have also been graded into four different grades depending upon the overall severity of the scarring present regardless of the individual morphology of the scars.[8] We have analyzed the efficacy of dermaroller, both in different types of scars and different grades of scars. Excellent response was seen in rolling or boxcar scars, while moderate response was seen in pitted scars. The severity of scars improved by two or more grades in 26 (72.2%) of our patients and in a further six (16.7%) patients we could achieve a reduction in scars by a single grade. Thus, on an overall basis a good to excellent response was achieved in 32 out of 36 patients (88.7%). It was also interesting to note that facial scars due to other etiologies also responded, with good or excellent response. Dermaroller treatment thus has definite advantages such as its cheaper cost, the comparative ease of the overall procedure and also the minimal downtime associated with it. Lack of any significant adverse effects is also an added advantage.

It is also important to emphasize the limitations of dermaroller treatment. Grade 4 scars did not respond as well as Grade 3 and Grade 2 scars. Some types of scars like linear scars or deep pitted scars did not respond well. However, these scars are difficult to treat even by other modalities such as lasers and may need surgical correction.

In summary, dermaroller is a simple, inexpensive office method of treatment for management of facial scars of different etiologies. It represents an important tool in the dermatosurgeon's armamentarium in managing this common and challenging cosmetic problem.
